# *RNF213* p.Arg4810Lys Wild Type is Associated with De Novo Hemorrhage in Asymptomatic Hemispheres with Moyamoya Disease

**DOI:** 10.1007/s12975-023-01159-z

**Published:** 2023-06-03

**Authors:** Seiei Torazawa, Satoru Miyawaki, Hideaki Imai, Hiroki Hongo, Daiichiro Ishigami, Masahiro Shimizu, Hideaki Ono, Yuki Shinya, Daisuke Sato, Yu Sakai, Motoyuki Umekawa, Satoshi Kiyofuji, Daisuke Shimada, Satoshi Koizumi, Daisuke Komura, Hiroto Katoh, Shumpei Ishikawa, Hirofumi Nakatomi, Akira Teraoka, Nobuhito Saito

**Affiliations:** 1 Department of Neurosurgery, Faculty of Medicine, The University of Tokyo, Tokyo, 113-8655 Japan; 2 Department of Neurosurgery, Tokyo Shinjuku Medical Center, Tokyo, Japan; 3 Department of Neurosurgery, Kanto Neurosurgical Hospital, Kumagaya, Japan; 4 Department of Neurosurgery, Fuji Brain Institute and Hospital, Fujinomiya, Japan; 5 Department of Neurosurgery, Kyorin University Hospital, Mitaka, Japan; 6 Department of Preventive Medicine, Graduate School of Medicine, The University of Tokyo, Tokyo, 113-8655 Japan; 7 Department of Neurosurgery, Teraoka Memorial Hospital, Fukuyama, Japan

**Keywords:** Moyamoya disease, *RNF213*, Genotype, Phenotype

## Abstract

Clinical implications of *RNF213* genetic variants, other than p.Arg4810Lys, in moyamoya disease (MMD), remain unclear. This study aimed to investigate the association of *RNF213* variants with clinical phenotypes in MMD. This retrospective cohort study collected data regarding the clinical characteristics of 139 patients with MMD and evaluated the angioarchitectures of 253 hemispheres using digital subtraction angiography at diagnosis. All *RNF213* exons were sequenced, and the associations of clinical characteristics and angiographical findings with p.Arg4810Lys, p.Ala4399Thr, and other rare variants (RVs) were examined. Among 139 patients, 100 (71.9%) had p.Arg4810Lys heterozygote (GA) and 39 (28.1%) had the wild type (GG). Fourteen RVs were identified and detetcted in 15/139 (10.8%) patients, and p.Ala4399Thr was detected in 17/139 (12.2%) patients. Hemispheres with GG and p.Ala4399Thr presented with significantly less ischemic events and more hemorrhagic events at diagnosis (*p* = 0.001 and *p* = 0.028, respectively). In asymptomatic hemispheres, those with GG were more susceptible to de novo hemorrhage than those with GA (adjusted hazard ratio [aHR] 5.36) with an increased risk when accompanied by p.Ala4399Thr or RVs (aHR 15.22 and 16.60, respectively). Within the choroidal anastomosis–positive hemispheres, GG exhibited a higher incidence of de novo hemorrhage than GA (*p* = 0.004). The GG of p. Arg4810Lys was a risk factor for de novo hemorrhage in asymptomatic MMD hemispheres. This risk increased with certain other variants and is observed in choroidal anastomosis–positive hemispheres. A comprehensive evaluation of *RNF213* variants and angioarchitectures is essential for predicting the phenotype of asymptomatic hemispheres in MMD.

**Supplementary Information:**

The online version contains supplementary material available at 10.1007/s12975-023-01159-z.

## Introduction

Moyamoya disease (MMD) is a rare cerebrovascular disorder characterized by progressive stenosis or occlusion of the terminal portion of the internal carotid artery (ICA). MMD leads to ischemic stroke including infarction and transient ischemic attack (TIA) because of reduced cerebral blood flow and hemorrhagic stroke because of the disruption of vulnerable collateral vessels [1–4]. The *RNF213* gene was identified in 2011 as a susceptibility gene for MMD, and *RNF213* c.14429G > A (p.Arg4810Lys, rs112735431) (based on NM_001256071 and NP_00124300 in the National Center for Biotechnology Information Reference Sequences) was found to be significantly associated with MMD [5, 6]. An association between the genotype of *RNF213* p.Arg4810Lys and the phenotype of MMD has been established, with the frequency of ischemic stroke being higher in heterozygous (GA) patients than in wild type (GG) patients [7–10]. However, the non-p.Arg4810Lys variants associated with the clinical presentation of patients have not yet been fully understood.

*RNF213* is a large gene with 68 exons that encodes 5207 amino acids, and many variants in coding exons other than p.Arg4810Lys have been reported in patients with MMD [4]. However, only a few of these variants are associated with the phenotype. Wu et al. reported that p.Ala4399Thr was associated with hemorrhage in Chinese patients with MMD [8], and Park et al. showed that p.Glu4950Asp was observed more frequently in ischemic MMD than in hemorrhagic MMD [11]. A few reports regarding other *RNF213* rare variants (RVs) have indicated that RVs are associated with clinical presentation in patients with MMD [12–14]. However, it remains unclear which *RNF213* variants, apart from p.Arg4810Lys, are associated with the development of ischemia or hemorrhage, despite these previous reports. Thus, additional investigations are needed to fully elucidate the relationship between the phenotype and the *RNF213* variants.

Clarifying angio architectural features such as periventricular anastomosis (PA) is crucial for analyzing the association between genotypes and phenotypes in patients with MMD. In particular, choroidal PA is widely recognized as a risk factor for hemorrhage [15–19]. However, the relationship between the *RNF213* variants and PA is not fully understood. Only a few studies on Chinese cohorts have demonstrated this association [20, 21], and no reports on Japanese cohorts have been published.

In this study, we aimed to identify indicators that could predict the hemispheric manifestation of MMD in terms of genetic and angiographical profiles by analyzing the association of phenotypes (ischemia or hemorrhage) and digital subtraction angiography (DSA) features of each hemisphere with the *RNF213* variants by sequencing all exons of *RNF213*. Additionally, we investigated the association between de novo cerebrovascular events in asymptomatic hemispheres and the genotype of *RNF213*.

## Methods

### Study Design and Participants

We conducted a retrospective cohort study using the data from patients with MMD and their hemispheres, collected according to the flowchart shown (Supplementary Fig. S1). We consecutively recruited 312 patients diagnosed with MMD who underwent blood sampling for genetic analysis at the University of Tokyo Hospital, Kanto Neurosurgical Hospital, Fuji Brain Institute and Hospital, and Teraoka Memorial Hospital between October 2011 and October 2022. The Human Genome Gene Analysis Research Ethics Committee of the Faculty of Medicine, University of Tokyo (approval number: G10026; approval date: September 12, 2011), and the Ethics Committees of Kanto Neurosurgical Hospital, Fuji Brain Institute and Hospital, and Teraoka Memorial Hospital approved this study. Written informed consent was obtained from all participants. Seventeen patients were diagnosed with quasi-MMD (see Supplementary Methods for the diagnostic criteria), six had previous revascularization surgeries at other institutions with insufficient preoperative data, and seven had insufficient clinical data; these patients were excluded. Among the 282 patients, we excluded 143 patients who did not undergo cerebral DSA at diagnosis. Finally, 139 patients who underwent DSA at the time of diagnosis were enrolled in the study. Since the clinical presentations and angiographical features often differ even between the hemispheres of identical patients, in this study, an analysis was performed per hemisphere. Among the 139 patients, 253 hemispheres were included in this study, excluding hemispheres with Suzuki grade 0. MMD was diagnosed based on the latest guidelines of the Research Committee on Moyamoya Disease and the Japan Stroke Society [22].

### Data Collection

Data were collected from medical records for the following parameters: age, sex, clinical presentation at diagnosis (ischemic or hemorrhagic onset, or asymptomatic in each hemisphere), de novo ischemic or hemorrhagic events in asymptomatic hemispheres, medical history (hypertension, diabetes mellitus, and dyslipidemia) (for the diagnostic criteria, see Supplementary Methods), smoking history (current smoker or not at diagnosis), family history of stroke and MMD, and angiographical profiles, as described below. Ischemic symptoms included cerebral infarction and TIA. Hemorrhagic symptoms included intracranial hemorrhage, intraventricular hemorrhage, and subarachnoid hemorrhage.

### Classification and Evaluation of Angiographical Features

PA, posterior cerebral artery (PCA) involvement, and Suzuki grade were evaluated utilizing cerebral DSA at diagnosis in 253 hemispheres. The definition and classification of PA were based on previous studies [23, 24]. In brief, PA was defined as “present” when there was a clear connection in the periventricular region between the perforating or choroidal arteries and the medullary or insular arteries. The anastomoses were classified into the following three subtypes: (1) lenticulostriate, beginning at the lenticulostriate artery; (2) choroidal, beginning at the anterior or posterior choroidal arteries; and (3) thalamic, beginning at the thalamotuberal, thalamogeniculate, or thalamoperforating arteries (Supplementary Fig. S2). PCA involvement was defined as occlusion or stenosis of >50% in segments P1–P3. DSA findings were assessed independently by two experienced neurosurgeons (S. T. and D. S.). The investigators were blinded to the patients’ genotypes and clinical information during the evaluation.

### Sequencing All Exons of *RNF213* and Genotyping of the *RNF213* Variants

The Twist Comprehensive Exome Panel Kit (South San Francisco, California, USA) was used to perform sequencing of all exons of *RNF213*. Sequencing data were generated using NovaSeq6000 (San Diego, California, USA) and the 150 basepair paired-end sequencing protocol across rapid-flow cell lanes. FastQC was used to ensure that the quality of all Fastq files was not classified as “fail.” Alignment to the human reference genome (Genome Reference Consortium Human Build 38 (GRCh38) [hg38]) and variant detection were performed using the Clara Parabricks 3.8.0 implementation of the Burrows–Wheeler Aligner and HaplotypeCaller, respectively. Passing variants annotated as PASS in the variant call format file were analyzed. Variants in the chr17:80260852 to chr17:80398794 (hg38) regions were extracted using bcftools to obtain the *RNF213* region.

All detected variants were checked against the Genome Browser and dbSNP databases (https://www.ncbi.nlm.nih.gov/snp) to obtain the rsIDs for each variant. The allele frequency of each variant was analyzed using the Genome Aggregation Database (gnomAD) (v.3.1.2) and a database from the Tohoku Medical Megabank Organization (ToMMo 14KJN). We used NM_001256071.3 (NP_001243000.2) as a reference sequence for *RNF213*, the major reference sequence for *RNF213* based on an experimentally verified open-reading frame by cDNA cloning [6].

First, the hotspot variant *RNF213* p.Arg4810Lys was evaluated. Moreover, RVs of *RNF213* were also used for the genotype–phenotype association study, following a previous study [20]. RVs were defined as those having a minor allele frequency <0.01 in both gnomAD and ToMMo. The deleteriousness of each variant was predicted using combined annotation-dependent depletion (GRCh38-v1.6 [The Genome Reference Consortium Human Genome Build 38]). Sorting intolerance from Tolerant and PolyPhen-2 were used to estimate the potential effects of amino acid substitutions.

A genotype–phenotype association study also evaluated two phenotype-associated variants, p.Ala4399Thr and p.Glu4950Asp. p.Ala4399Thr was reported to be associated with hemorrhagic MMD in China (odds ratio [OR] = 2.8) [8], while p.Glu4950Asp was observed more frequently in ischemic MMD than in hemorrhagic MMD in Korea (OR = 2.2) [11].

### Statistical Analysis

All statistical analyses were performed using the SPSS Statistics version 26 software (IBM Corp., Armonk, NY, USA). The kappa statistic (*κ*) was used to assess the interrater agreement on the presence of each PA. Mann–Whitney *U* tests were used to compare the proportions between groups for continuous data. The chi-square test or Fisher’s exact test was used for categorical variables to compare proportions. Logistic regression analysis was used for the multivariate analysis. For time-series data, Kaplan–Meier curves were generated, log-rank testing was used to determine *p-*values, and the Cox proportional hazard model was used to calculate the hazard ratio (HR) and adjusted HR (aHR) for multivariable adjustment. The person-years method was used to calculate the annual incidence of de novo ischemia and hemorrhage per hemisphere. Statistical significance was defined as *p* < 0.05.

## Results

One hundred and one (72.7%) of the 139 enrolled patients were female, with a mean age of 43 years (interquartile range, 35.5–52 years) (Table 1).

**Table 1:** Basic characteristics and clinical manifestations at diagnosis of each genotype of *RNF213* p.Arg4810Lys in all 139 cases

	All patients (*n* = 139)		*RNF213* wild type (GG) (*n* = 39)		*RNF213* heterozygote (GA) (*n* = 100)		*P* value
	*n*	%	*n*	%	*n*	%	
Female	101	72.7	29	74.4	72	72.0	0.779
Age at diagnosis, median (IQR)	43 (35.5–52)		44 (37–51)		43 (34.8–52.5)		0.739
Hypertension	62	44.6	17	43.6	45	45.0	0.881
Diabetes mellitus	11	7.9	4	10.3	7	7.0	0.371
Dyslipidemia	27	19.4	8	20.5	19	19.0	0.839
Current smoker	25	18.0	8	20.5	17	17.0	0.628
Family history of any stroke	33	23.7	5	12.8	28	28.0	0.059
Family history of MMD	26	18.7	3	7.7	23	23.0	**0.038**
Suzuki grade	3 (3–3)		3 (3–3)		3 (3–3)		0.674
Symptoms at diagnosis							
Asymptomatic	23	16.5	8	20.5	15	15.0	0.432
Ischemia	92	65.5	22	56.4	69	69.0	0.161
Hemorrhage	26	18.7	9	23.1	17	17.0	0.409
*RNF213* variants							
p.Ala4399Thr	17	12.2	9	23.1	8	8.0	**0.021**
p.Glu4950Asp	1	0.7	1	2.6	0	0.0	0.281
RVs	15	10.8	10	25.6	5	5.0	**0.001**

*IQR*, interquartile range; *MMD*, moyamoya disease; *RV*, rare variant

Values in bold indicate *p* < 0.05

Excluding the 25 hemispheres with a Suzuki grade of 0, 124 (49.0%) of the 253 hemispheres were asymptomatic, 103 (40.7%) were ischemic, and 26 (10.3%) were hemorrhagic at diagnosis. The frequencies of each subtype of PA were as follows: lenticulostriate, 58 hemispheres (22.9%); choroidal, 88 hemispheres (34.8%); and thalamic, 24 hemispheres (9.5%). PCA involvement was detected in 34 (13.4 %) hemispheres. The median Suzuki grade was 3 (Table 2).

**Table 2:** Clinical manifestations at diagnosis and angiographic features of each genetic variation in *RNF213* in 253 hemispheres

	All hemispheres (n = 253)		GA (*n* = 179)		GG (*n* = 74)			GG/p.Ala4399Thr (*n* = 17)			GG/RV (*n* = 19)		
	*n*	%	*n*	%	*n*	%	*P* value vs. GA	n	%	*P* value vs. GA	n	%	*P* value vs. GA
Symptoms at diagnosis													
Asymptomatic	124	49.0	83	46.4	41	55.4	0.191	11	64.7	0.148	9	47.4	0.934
Ischemia	103	40.7	79	44.1	24	32.4	0.085	1	5.9	**0.001**	8	42.1	0.865
Hemorrhage	26	10.3	17	9.5	9	12.2	0.525	5	29.4	**0.028**	2	10.5	0.568
Angiographical features													
Lenticulostriate PA	58	22.9	52	29.1	6	8.1	**<0.001**	1	5.9	**0.029**	2	10.5	0.085
Choroidal PA	88	34.8	64	35.8	24	32.4	0.614	6	35.3	0.970	6	31.6	0.717
Thalamic PA	24	9.5	22	12.3	2	2.7	**0.018**	1	5.9	0.379	0	0.0	0.095
PCA involvement	34	13.4	28	15.6	6	8.1	0.110	0	0.0	0.064	3	15.8	0.600
Suzuki grade, median (IQR)	3 (3–3)		(3–3)		3 (3–3)		0.474	3 (3–3)		0.323	3 (3–3)		0.561

*GA*, heterozygote of p.Arg4810Lys; *GG*, wild type of p.Arg4810Lys; *PA*, periventricular anastomosis, *PCA*; posterior cerebral artery; *RV*, rare variant

Values in bold indicate *p* < 0.05

### Reliability of Evaluation of PA

Interrater reliability for the presence of each subtype of PA was almost perfect (lenticulostriate, κ = 0.883; choroidal, κ = 0.903; thalamic, κ = 0.801). When there was a disagreement among the raters’ assessments, the evaluations were determined through discussion with the patient's genotype or other relevant clinical data blinded.

### Genotype of *RNF213*

*RNF213* p.Arg4810Lys was present in 100 patients (71.9%); all were heterozygous (GA), and none were homozygous. Sequencing of whole exons of *RNF213*, 14 RVs were identified in 15 patients (10.8%), including p.His119Tyr, p.Pro253Ser, p.His443Asp, p.Arg1023Trp, p.Asp2007Asn, p.Gly2440Asp, p.Arg2704Gln, p.Arg2709Thr, p.Glu3061Lys, p.Met3666Thr, p.Val4015Met, p.Pro4250Thr, p.Glu4950Asp, and p.Ser5083Ala. Information for each variant is listed in Supplementary Table S1. Five (5.0%) of the 100 patients with GA had RVs, and 10 (25.6%) of the 39 patients with GG had RVs (*p* = 0.001) (Table 1).

As for the phenotype-associated variants, p.Glu4950Asp was found in only one patient (0.7%), whereas p.Ala4399Thr was detected in 17 patients (12.2%). p.Ala4399Thr was more frequent among patients with GG than among those with GA (*p* = 0.021) (Table 1). Nine (52.9%) of the 17 patients with p.Ala4399Thr were ischemic, and seven (41.2%) were hemorrhagic. The patient with the p.Glu4950Asp variant had ischemia (Supplementary Table S2).

### Association of *RNF213* Variants with Phenotype at Diagnosis and Angiographical Features

We analyzed the association between genotype, clinical manifestations at diagnosis, and angiographical features for each hemisphere.

At the time of diagnosis, nine (29.0%) of the 31 hemispheres with p.Ala4399Thr were ischemic and five (16.1%) were hemorrhagic. Regarding the RV, 13 (46.4%) of the 28 hemispheres with RV were ischemic and 3 (10.7%) were hemorrhagic at diagnosis. The presence of p.Ala4399Thr or RV was not associated with symptoms at diagnosis or angiographical features (Supplementary Table S3).

Next, we analyzed the GG with p.Ala4399Thr group (GG/p. Ala4399Thr, 17 hemispheres) and GG with RV group (GG/RV, 19 hemispheres) to examine the effect of p.Ala4399Thr or RV on GG cases: Three groups, the GG, GG/p. Ala4399Thr, and GG/RV, were compared with GA (Table 2).

Regarding the angiographical features at diagnosis, lenticulostriate PA (GG, 8.1%; GA, 29.1%) and thalamic PA (GG, 2.7%; GA, 12.3%) were significantly more common in the GA group than in the GG group (*p* < 0.001 and *p* = 0.018, respectively). There were no significant differences in choroidal PA between the genotypes. Notably, regarding symptoms at diagnosis, the GG/p.Ala4399Thr group presented with significantly less ischemia and more hemorrhage (*p* = 0.001 and *p* = 0.028, respectively). In the analysis of the association between angiographical features and clinical manifestations at diagnosis, choroidal PA was significantly associated with hemorrhagic onset (*p* = 0.010), and Suzuki grade was significantly associated with both ischemic and hemorrhagic onset hemispheres (*p* = 0.002 and *p* = 0.042, respectively) (Supplementary Table S4).

The adjusted odd ratios (aORs) of each genotype for ischemic or hemorrhagic onset were calculated using multivariate logistic regression models. After correcting for age, sex, and Suzuki grade with the ischemic onset and sex, age, Suzuki grade, and choroidal PA with hemorrhagic onset, significant associations remained in the GG/p.Ala4399Thr group: significantly less ischemia and more hemorrhage (*p* = 0.019, aOR 0.08, 95% confidence interval [CI] 0.01–0.67, and *p* = 0.021, aOR 4.59, 95% CI 1.26–16.72, respectively) (Table 3).

**Table 3:** Adjusted odds ratios of each genotype group for ischemic or hemorrhagic onset compared with *RNF213* p.Arg4810Lys heterozygotes

		*P* value	aOR (95% CI)
Ischemic onset (adjusted for age, sex, and Suzuki grade)			
GG	(vs. GA)	0.123	0.63 (0.35–1.13)
GG/p.Ala4399Thr	(vs. GA)	**0.019**	**0.08 (0.01–0.67)**
GG/RV	(vs. GA)	0.774	1.16 (0.42–3.21)
Hemorrhagic onset (adjusted for age, sex, Suzuki grade, and choroidal PA)			
GG	(vs. GA)	0.381	1.50 (0.61–3.70)
GG/p.Ala4399Thr	(vs. GA)	**0.021**	**4.59 (1.26–16.72)**
GG/RV	(vs. GA)	0.921	1.09 (0.21–5.53)

*aOR*, adjusted odds ratio; *CI*, confidence interval; *GA*, heterozygote of p.Arg4810Lys; *GG*, wild type of p.Arg4810Lys; *PA*, periventricular anastomosis; *RV*, rare variant

Values in bold are statistically significant *p* < 0.05

### Analysis of De Novo Ischemia/Hemorrhage in Asymptomatic Hemispheres

Furthermore, we analyzed the occurrence of de novo cerebrovascular events (ischemia or hemorrhage) in the asymptomatic hemispheres (*n* = 122; two cases were excluded because of a lack of follow-up data). The mean follow-up duration for the de novo event was 6.7 years. The annual incidences of de novo ischemia (*n* = 15 hemispheres, 12.3%) and hemorrhage (*n* = 15 hemispheres, 12.3%) in asymptomatic hemispheres were 1.8% per hemisphere.

Risk factors for atherosclerosis (HT, DM, HL, and smoking), angiographical features, and genotype groups (GG, GG/p.Ala4399Thr, and GG/RV) were examined for their association with de novo ischemia or hemorrhage. The results of the log-rank tests are presented in Supplementary Fig. S3.

**Fig. 1:**
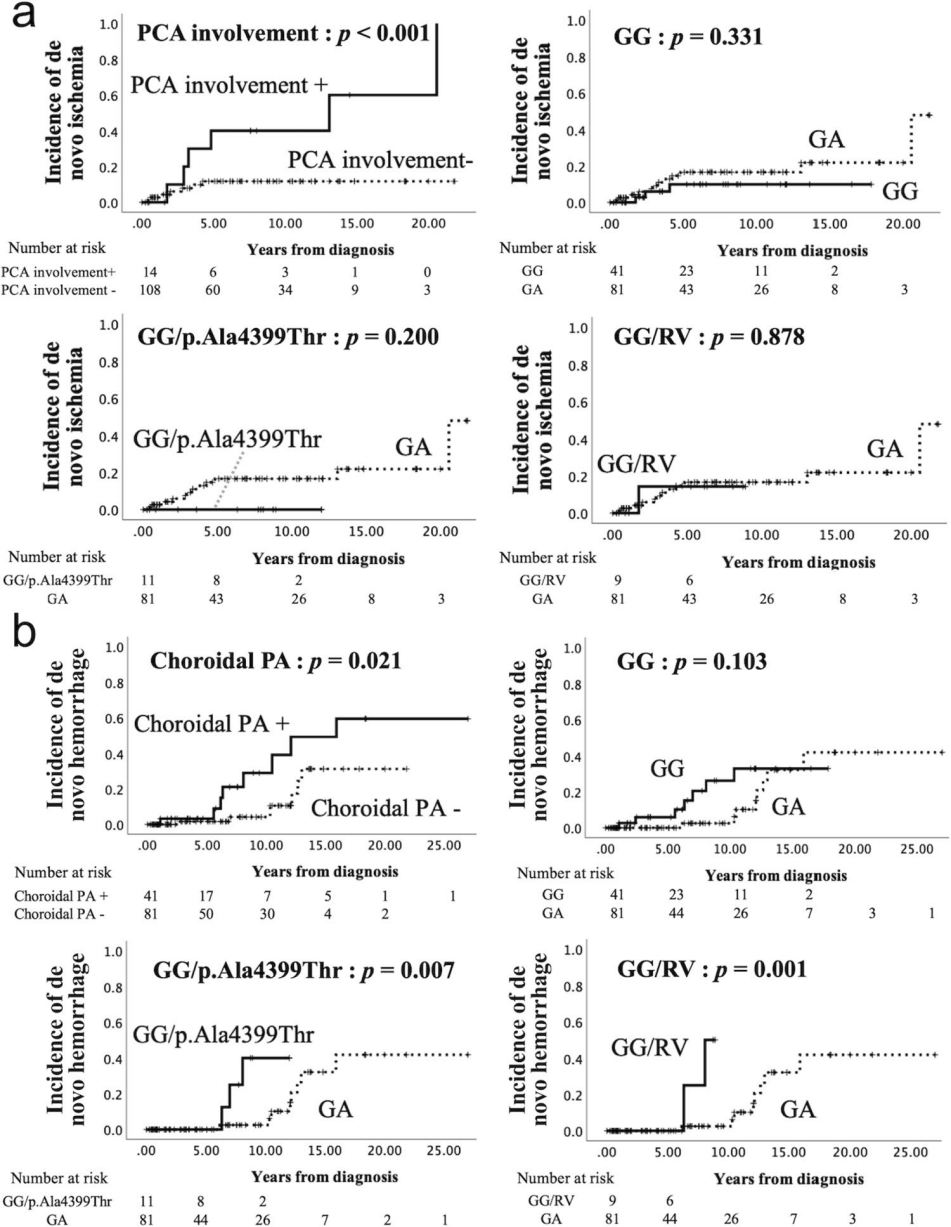
Kaplan–Meier curves for de novo ischemia and hemorrhage in asymptomatic hemispheres. Kaplan–Meier curves for de novo ischemia (**a**) and de novo hemorrhage (**b**) in the asymptomatic hemispheres. PCA involvement is a risk factor for de novo ischemia in terms of angiographical features, whereas choroidal PA is associated with de novo hemorrhage. GG/p.Ala4399Thr and GG/RV were significant risk factors for de novo hemorrhage compared to GA regarding the genotype groups. Kaplan–Meier curves analyzed by the other angiographical features and other factors (sex, HT, DM, HL, and smoking) are shown in Supplementary Fig. S3. *P*-values were calculated using the log-rank tests. GA, heterozygote of p.Arg4810Lys; GG, wild type of p.Arg4810Lys; PA, periventricular anastomosis; PCA, posterior cerebral artery; RV, rare variant

No atherosclerotic risk factors were significantly associated with de novo ischemia or hemorrhage. PCA involvement was significantly related to de novo ischemia (*p* < 0.001, HR 6.23, 95% CI 2.23–17.42), whereas choroidal PA was associated with de novo hemorrhage (*p* = 0.021, HR 3.16, 95% CI 1.13–8.85). The annual incidence of de novo ischemia in PCA-involvement–positive asymptomatic hemispheres was 7.7% per hemisphere and that of de novo hemorrhage in choroidal PA–positive asymptomatic hemispheres was 3.4% per hemisphere. GG/p.Ala4399Thr and GG/RV were significantly associated with de novo hemorrhage (*p* = 0.007, HR 6.94, 95% CI 1.35–35.58 and *p* = 0.001, HR 18.442, 95% CI 1.67–203.91, respectively) regarding the genotype groups. Annual incidences of de novo hemorrhage in these two groups were 3.9% per hemisphere and 4.2% per hemisphere, respectively. The Kaplan–Meier curves for the angiographical features that demonstrated significant associations and those for each genotype group are depicted in Fig. 1. The RV of the two de novo hemorrhagic cases in the GG/RV group were p.P4250T.

Finally, the aHR of each genotype group for de novo ischemia or hemorrhage compared to GA was calculated using the Cox proportional hazard model, with correction for age, sex, Suzuki grade, and statistically significant angiographical factors for each ischemia or hemorrhage. Even after adjustment, GG and GG/p.Ala4399Thr and GG/RV were more susceptible to de novo hemorrhage than GA (*p* = 0.009, aHR 5.36, 95% CI 1.53–18.85; *p* = 0.012, aHR 15.22, 95% CI 1.82–127.28; and *p* = 0.034, aHR 16.60, 95% CI 1.24–222.84, respectively) (Table 4).

**Table 4:** Risk genotypes for de novo ischemia or hemorrhage in asymptomatic hemispheres compared to *RNF213* p.Arg4810Lys heterozygote (after adjustment for angiographic profiles)

		*P* value	aHR	95% CI
De novo ischemia (adjusted for age, sex, Suzuki grade, and PCA involvement)				
GG	(vs. GA)	0.924	0.94	0.23–3.75
GG/p.Ala4399Thr	(vs. GA)	0.984	0	NA
GG/RV	(vs. GA)	0.52	2.02	0.23–17.44
De novo hemorrhage (adjusted for age, sex, Suzuki grade, and choroidal PA)				
GG	(vs. GA)	**0.009**	**5.36**	**1.53–18.85**
GG/p.Ala4399Thr	(vs. GA)	**0.012**	**15.22**	**1.82–127.28**
GG/RV	(vs. GA)	**0.034**	**16.60**	**1.24–222.84**

*aHR*, adjusted hazard ratio; *CI*, confidence interval; *GA*, heterozygote of p.Arg4810Lys; *GG*, wild type of p.Arg4810Lys; *PA*, periventricular anastomosis, *PCA*; posterior cerebral artery; *RV*, rare variant

Values in bold are statistically significant *p* < 0.05

### Subgroup Analysis for De novo Hemorrhage by Presence/Absence of Choroidal PA

**Fig. 2:**
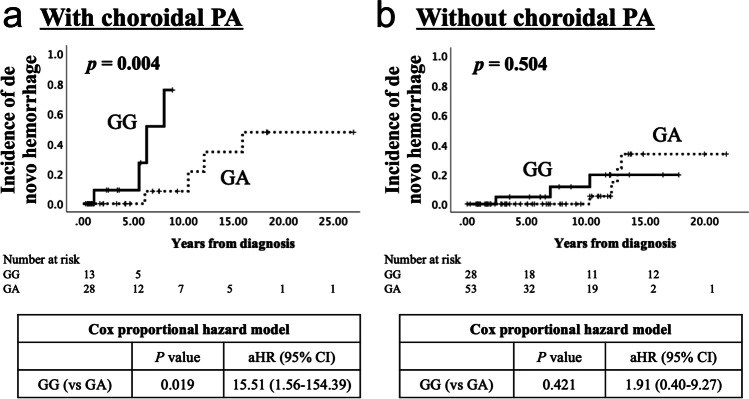
Kaplan–Meier curves for de novo hemorrhage in asymptomatic hemispheres with or without choroidal periventricular anastomosis. Kaplan–Meier curves for de novo hemorrhage in asymptomatic hemispheres with (**a**) or without (**b**) choroidal PA. The log-rank test revealed that GG was significantly more susceptible to de novo hemorrhage than GA within the hemispheres with choroidal PA. Conversely, there was no significant difference by genotype in the hemispheres without PA. aHR, adjusted hazard ratio; CI, Confidence interval; GA, heterozygote of p.Arg4810Lys; GG, wild type of p.Arg4810Lys; PA, periventricular anastomosis

Choroidal PA was a major risk factor for hemorrhage, as previously reported, and the frequency of choroidal PA was approximately the same regardless of the genotype. However, susceptibility to hemorrhage in the hemispheres with GG was observed in this study. Therefore, we performed a subgroup analysis of hemispheres with or without choroidal PA to elucidate whether susceptibility to hemorrhage in hemispheres with choroidal PA differed by genotype. The result was that GG was significantly more prone to de novo hemorrhage among the hemispheres with choroidal PA than was GA (*p* = 0.004, HR 12.50, 95% CI 1.38–113.54) (Fig. 2). After adjusting for age and sex using the Cox proportional hazard model, this association remained statistically significant (*p* = 0.019, HR 15.51, 95% CI 1.56–154.39).

## Discussion

In this study, we identified the significance of the GG of p.Arg4810Lys and other *RNF213* variants in asymptomatic hemispheres by sequencing all exons of *RNF213*. We also revealed an association between *RNF213* variants and the development of PA, indicating that the clinical course may vary depending on p.Arg4810Lys in hemispheres with choroidal PA.

Our study showed that the GG of p.Arg4810Lys was more susceptible to de novo hemorrhage in asymptomatic hemispheres than was the GA, although the definitive genetic factors that determine various clinical presentations in patients with MMD have not been conclusively clarified [25]. The risk increased with the presence of p.Ala4399Thr or RVs.

Among the various *RNF213* variants, p.Ala4399Thr is the only variant reported to be associated with hemorrhage in patients with MMD, and Wu et al. reported that p.Ala4399Thr was associated with MMD (OR = 2.0), especially hemorrhagic MMD (OR = 2.8), in a Chinese population [8]. Kobayashi et al. reported a case of pulmonary hypertension with the p.Ala4399Thr variant, suggesting that p.Ala4399Thr may be involved in vascular abnormalities [26]. Based on these reports and in silico predictions of the pathogenicity of this variant (Supplementary Table S2), it is possible that p.Ala4399Thr acts as a modifier of the MMD phenotype.

Other various *RNF213* RVs have also been documented in MMD [4, 25]. Regarding the association between *RNF213* RVs and the clinical phenotypes of patients with MMD, various studies have reported an association between these variants and the infantile or early onset of MMD [12–14]. However, to date, no report has established a relationship between RVs and ischemic or hemorrhagic manifestations of MMD. This study indicates that asymptomatic hemispheres with GG and RV could be at a higher risk of de novo hemorrhage. The two hemispheres with GG and RV that experienced de novo hemorrhage had a common variant, p.Pro4250Thr. This variant has not been previously documented in patients with MMD. The functional effect of p.Pro4250Thr was assumed to be relatively non-deleterious (Supplementary Table S1). However, this variant is located in the C-terminal region of *RNF213*, similar to p.Arg4810Lys and p.Ala4399Thr. The C-terminal region of *RNF213* encompasses the RING-finger domain, and RING-finger proteins have been reported to act as E3 ubiquitin ligases [27]. Guey et al. reported that rare *RNF213* variants associated with Caucasian patients with MMD were preferentially located in this region. Regarding the importance of the C-terminal variants of *RNF213*, they showed that a change in the *RNF213* RING-finger structure or function might play a critical role in moyamoya pathogenesis [13]. Hence, we propose that p.Ala4399Thr and p.Pro4250Thr may be pathogenic because they alter the function of E3 ubiquitin ligases, leading to the clinical presentation of MMD.

When considering choroidal PA, a well-known risk factor for hemorrhage in MMD [15–19], there have only been a few subsequent reports from China regarding the association between choroidal PA and *RNF213* variants. Xue et al. showed that choroidal PA significantly developed in the presence of the p.Arg4810Lys variant, and other types of PA also significantly developed with both p.Arg4810Lys and other RVs [20]. Ge et al. revealed that the p.Arg4810Lys variant was significantly associated with the development of lenticulostriate PA in the hemorrhagic hemispheres of Chinese patients with MMD [21]. The prevalence of the p.Arg4810Lys variant has been reported to differ considerably between China and Japan [5, 8, 28–31], and racial differences in PA development have been documented [32]. This is the first report to analyze the association between PA and *RNF213* variants in a Japanese cohort. This study demonstrated that the p.Arg4810Lys variant was significantly associated with the development of lenticulostriate and thalamic PA, whereas the development of choroidal PA was not associated with any genotype.

Our subgroup analysis revealed that susceptibility to de novo hemorrhage varied among hemispheres with choroidal PA, depending on the presence of p.Arg4810Lys. This result might be explained by the relationship between PA and p.Arg4810Lys mentioned above. Based on our finding that lenticulostriate and thalamic PA in the hemispheres with GG were less developed than those in the hemispheres with GA, we hypothesized that the blood flow of the ICA would be concentrated in the choroidal artery in the GG hemisphere, resulting in an enormous hemodynamic burden and eventual rupture. We need to monitor the clinical course of more choroidal PA–positive asymptomatic hemispheres to validate this hypothesis further.

In the context of the clinical implications of the genetic diagnosis of MMD, most previous studies have focused on *RNF213* p.Arg4810Lys. They have demonstrated its clinical associations, including its association with earlier onset [7, 33], ischemic onset [7–9, 33], postoperative collateral formation [34–36], and the functional effectiveness of revascularization surgery [7, 35]. In contrast, our study sheds light on the clinical implications of the GG of p.Arg4810Lys and other variants such as p.Ala4399Thr or *RNF213* RVs. Based on our findings, we contend that identifying p.Arg4810Lys and other *RNF213* variants by sequencing all exons of *RNF213* is essential for accurately predicting the clinical course of MMD. Clinically, we recommend closely monitoring asymptomatic hemispheres with GG, particularly those accompanied by p.Ala4399Thr, other RVs, or choroidal PA. If such asymptomatic hemispheres are present, intensified control of general cardiovascular risk factors or early revascularization surgery may be the optimal approaches to prevent hemorrhagic events.

This study has some limitations. First, this was a retrospective cohort study, and we did not consecutively enroll all the patients diagnosed with MMD. Only patients who underwent DSA at diagnosis were selected; therefore, a selection bias existed. Second, the sample size was modest. The number of RVs detected in this study was limited. Third, the pathophysiological mechanism by which variants associated with hemorrhage cause bleeding has not been elucidated. Further accumulation of cases and experimental research on these variants is needed to clarify the pathophysiological mechanism of the clinical manifestations of MMD.

## Conclusion

In this study, we elucidated that the GG of p.Arg4810Lys was a risk factor for de novo hemorrhage in asymptomatic hemispheres of patients with MMD, with a further increased risk when accompanied by p.Ala4399Thr or RVs. Furthermore, we demonstrated for the first time that susceptibility to de novo hemorrhage varies according to the p. Arg4810Lys genotype within hemispheres with choroidal PA. Therefore, a comprehensive evaluation of exonic variants of the whole *RNF213* and an accurate assessment of angiographical features are crucial for predicting the phenotype of asymptomatic hemispheres in MMD.

### Supplementary Information

ESM 1 Online Resource:Supplementary Methods. Supplementary Tables S1–S4. Supplementary Figures S1–S3. Supplementary Table S1 Characteristics of each identified rare variant of *RNF213* in this study. Supplementary Table S2 Information on *RNF213* p.Ala4399Thr and p.Glu4950Asp. Supplementary Table S3 Clinical manifestations at diagnosis and angiographic features of each hemisphere with p.Ala4399Thr or RV. Supplementary Table S4 Association between angiographic profiles and clinical manifestations at diagnosis. Supplementary Fig. S1 Flowchart of the selection process. Supplementary Fig. S2 Representative angiography of each type of periventricular anastomosis (PA). Supplementary Fig. S3 Kaplan–Meier curves for de novo ischemia and hemorrhage in asymptomatic hemispheres (DOCX 2114 kb)

## Data Availability

The data supporting the findings of this study are available from the corresponding author upon reasonable request from any investigator.

## References

[1] Suzuki J, Takaku A (1969). Cerebrovascular “moyamoya” disease. Disease showing abnormal net-like vessels in base of brain. Arch Neurol..

[2] Kuroda S, Houkin K (2008). Moyamoya disease: current concepts and future perspectives. Lancet Neurol..

[3] Scott RM, Smith ER (2009). Moyamoya disease and moyamoya syndrome. N Engl J Med..

[4] Ihara M, Yamamoto Y, Hattori Y, Liu W, Kobayashi H, Ishiyama H, Yoshimoto T, Miyawaki S, Clausen T, Bang OY, Steinberg GK, Tournier-Lasserve E, Koizumi A (2022). Moyamoya disease: diagnosis and interventions. Lancet Neurol..

[5] Kamada F, Aoki Y, Narisawa A, Abe Y, Komatsuzaki S, Kikuchi A, Kanno J, Niihori T, Ono M, Ishii N, Owada Y, Fujimura M, Mashimo Y, Suzuki Y, Hata A, Tsuchiya S, Tominaga T, Matsubara Y, Kure S (2011). A genome-wide association study identifies RNF213 as the first moyamoya disease gene. J Hum Genet..

[6] Liu W, Morito D, Takashima S, Mineharu Y, Kobayashi H, Hitomi T, Hashikata H, Matsuura N, Yamazaki S, Toyoda A, Kikuta K, Takagi Y, Harada KH, Fujiyama A, Herzig R, Krischek B, Zou L, Kim JE, Kitakaze M, Miyamoto S, Nagata K, Hashimoto N, Koizumi A (2011). Identification of RNF213 as a susceptibility gene for moyamoya disease and its possible role in vascular development. PLoS One..

[7] Nomura S, Yamaguchi K, Akagawa H, Kawashima A, Moteki Y, Ishikawa T, Aihara Y, Saito T, Okada Y, Kawamata T (2019). Genotype-phenotype correlation in long-term cohort of Japanese patients with moyamoya disease. Cerebrovasc Dis..

[8] Wu Z, Jiang H, Zhang L, Xu X, Zhang X, Kang Z, Song D, Zhang J, Guan M, Gu Y (2012). Molecular analysis of RNF213 gene for moyamoya disease in the Chinese Han population. PLoS One..

[9] Wang Y, Zhang Z, Wang X, Zou Z, Ta N, Hao F, Yang Y, Li D, Liang M, Han C, Bao X, Ou L, Wang H, Yang Z, Yang R, Zeng F, Shang M, Nie F, Liu W, Duan L (2021). Validation and extension study exploring the role of RNF213 p.R4810K in 2,877 Chinese moyamoya disease patients. J Stroke Cerebrovasc Dis..

[10] Wang Y, Yang L, Wang X, Zeng F, Zhang K, Zhang Q, Liu M, Liu S, Shang M, Li Q, Yang Y, Liang M, Liu W (2021). Meta-analysis of genotype and phenotype studies to confirm the predictive role of the RNF213 p.R4810K variant for moyamoya disease. Eur J Neurol..

[11] Park YS, An HJ, Kim JO, Kim WS, Han IB, Kim OJ, Kim NK, Kim DS (2017). The role of RNF213 4810G>A and 4950G>A variants in patients with moyamoya disease in Korea. Int J Mol Sci..

[12] Moteki Y, Onda H, Kasuya H, Yoneyama T, Okada Y, Hirota K, Mukawa M, Nariai T, Mitani S, Akagawa H (2015). Systematic validation of RNF213 coding variants in Japanese patients with moyamoya disease. J Am Heart Assoc..

[13] Guey S, Kraemer M, Hervé D, Ludwig T, Kossorotoff M, Bergametti F, Schwitalla JC, Choi S, Broseus L, Callebaut I, Genin E, Tournier-Lasserve E, FREX consortium. (2017). Rare RNF213 variants in the C-terminal region encompassing the RING-finger domain are associated with moyamoya angiopathy in Caucasians. Eur J Hum Genet..

[14] Hara S, Mukawa M, Akagawa H, Thamamongood T, Inaji M, Tanaka Y, Maehara T, Kasuya H, Nariai T (2022). Absence of the RNF213 p.R4810K variant may indicate a severe form of pediatric moyamoya disease in Japanese patients. J Neurosurg Pediatr..

[15] Morioka M, Hamada J, Kawano T, Todaka T, Yano S, Kai Y, Ushio Y (2003). Angiographic dilatation and branch extension of the anterior choroidal and posterior communicating arteries are predictors of hemorrhage in adult moyamoya patients. Stroke..

[16] Liu W, Zhu S, Wang X, Yue X, Zhou Z, Wang H, Xu G, Zhou C, Liu X (2011). Evaluation of angiographic changes of the anterior choroidal and posterior communicating arteries for predicting cerebrovascular lesions in adult moyamoya disease. J Clin Neurosci..

[17] Yamamoto S, Hori S, Kashiwazaki D, Akioka N, Kuwayama N, Kuroda S (2018). Longitudinal anterior-to-posterior shift of collateral channels in patients with moyamoya disease: an implication for its hemorrhagic onset. J Neurosurg..

[18] Fujimura M, Funaki T, Houkin K, Takahashi JC, Kuroda S, Tomata Y, Tominaga T, Miyamoto S (2019). Intrinsic development of choroidal and thalamic collaterals in hemorrhagic-onset moyamoya disease: case-control study of the Japan Adult Moyamoya Trial. J Neurosurg..

[19] Liu P, Liu AH, Han C, Chen C, Lv XL, Li DS, Ge HJ, Jin HW, Li YX, Duan L (2016). Difference in angiographic characteristics between hemorrhagic and nonhemorrhagic hemispheres associated with hemorrhage risk of moyamoya disease in adults: a self-controlled study. World Neurosurg..

[20] Xue Y, Zeng C, Ge P, Liu C, Li J, Zhang Y, Zhang D, Zhang Q, Zhao J (2022). Association of RNF213 variants with periventricular anastomosis in moyamoya disease. Stroke..

[21] Ge P, Zhang Q, Ye X, Liu X, Deng X, Wang J, Wang R, Zhang Y, Zhang D, Zhao J (2020). Different subtypes of collateral vessels in hemorrhagic moyamoya disease with p.R4810K variant. BMC Neurol..

[22] Kuroda S, Fujimura M, Takahashi J, Kataoka H, Ogasawara K, Iwama T, Tominaga T, Miyamoto S (2022). Diagnostic criteria for moyamoya disease - 2021 revised version. Neurol Med Chir (Tokyo)..

[23] Funaki T, Fushimi Y, Takahashi JC, Takagi Y, Araki Y, Yoshida K, Kikuchi T, Miyamoto S (2015). Visualization of periventricular collaterals in moyamoya disease with flow-sensitive black-blood magnetic resonance angiography: preliminary experience. Neurol Med Chir (Tokyo)..

[24] Funaki T, Takahashi JC, Yoshida K, Takagi Y, Fushimi Y, Kikuchi T, Mineharu Y, Okada T, Morimoto T, Miyamoto S (2016). Periventricular anastomosis in moyamoya disease: detecting fragile collateral vessels with MR angiography. J Neurosurg..

[25] Mertens R, Graupera M, Gerhardt H, Bersano A, Tournier-Lasserve E, Mensah MA, Mundlos S, Vajkoczy P (2022). The genetic basis of moyamoya disease. Transl Stroke Res..

[26] Kobayashi H, Kabata R, Kinoshita H, Morimoto T, Ono K, Takeda M, Choi J, Okuda H, Liu W, Harada KH, Kimura T, Youssefian S, Koizumi A (2018). Rare variants in RNF213, a susceptibility gene for moyamoya disease, are found in patients with pulmonary hypertension and aggravate hypoxia-induced pulmonary hypertension in mice. Pulm Circ..

[27] Deshaies RJ, Joazeiro CA (2009). RING domain E3 ubiquitin ligases. Annu Rev Biochem..

[28] Miyatake S, Miyake N, Touho H, Nishimura-Tadaki A, Kondo Y, Okada I, Tsurusaki Y, Doi H, Sakai H, Saitsu H, Shimojima K, Yamamoto T, Higurashi M, Kawahara N, Kawauchi H, Nagasaka K, Okamoto N, Mori T, Koyano S, Kuroiwa Y, Taguri M, Morita S, Matsubara Y, Kure S, Matsumoto N (2012). Homozygous c.14576G>A variant of RNF213 predicts early-onset and severe form of moyamoya disease. Neurology..

[29] Miyawaki S, Imai H, Shimizu M, Yagi S, Ono H, Mukasa A, Nakatomi H, Shimizu T, Saito N (2013). Genetic variant RNF213 c.14576G>A in various phenotypes of intracranial major artery stenosis/occlusion. Stroke..

[30] Wang Y, Zhang Z, Wei L, Zhang Q, Zou Z, Yang L, Li D, Shang M, Han C, Mambiya M, Bao X, Li Q, Hao F, Zhang K, Wang H, Liu S, Liu M, Zeng F, Nie F, Wang K, Liu W, Duan L (2020). Predictive role of heterozygous p.R4810K of RNF213 in the phenotype of Chinese moyamoya disease. Neurology..

[31] Zhang Q, Liu Y, Zhang D, Wang R, Zhang Y, Wang S, Yu L, Lu C, Liu F, Zhou J, Zhang X, Zhao J (2017). RNF213 as the major susceptibility gene for Chinese patients with moyamoya disease and its clinical relevance. J Neurosurg..

[32] Hori S, Kashiwazaki D, Yamamoto S, Acker G, Czabanka M, Akioka N, Kuwayama N, Vajkoczy P, Kuroda S (2019). Impact of interethnic difference of collateral angioarchitectures on prevalence of hemorrhagic stroke in moyamoya disease. Neurosurgery..

[33] Ge P, Ye X, Liu X, Deng X, Wang R, Zhang Y, Zhang D, Zhang Q, Zhao J (2019). Association between p. R4810K variant and long-term clinical outcome in patients with moyamoya disease. Front Neurol..

[34] Ge P, Ye X, Liu X, Deng X, Wang J, Wang R, Zhang Y, Zhang D, Zhang Q, Zhao J (2019). Association between p. R4810K variant and postoperative collateral formation in patients with moyamoya disease. Cerebrovasc Dis..

[35] Zhang Q, Ge P, Ma Y, Zhang D, Wang R, Zhang Y, Wang S, Cao Y, Zhao M, Zhao J (2019). Clinical features and surgical outcomes of patients with moyamoya disease and the homozygous RNF213 p.R4810K Variant. J Child Neurol..

[36] Ito M, Kawabori M, Sugiyama T, Tokairin K, Tatezawa R, Uchino H, Kazumata K, Houkin K, Fujimura M (2022). Impact of RNF213 founder polymorphism (p.R4810K) on the postoperative development of indirect pial synangiosis after direct/indirect combined revascularization surgery for adult moyamoya disease. Neurosurg Rev..

