# Supplementary material for: RNF213 p.Arg4810Lys Wild Type is Associated with De Novo Hemorrhage in Asymptomatic Hemispheres with Moyamoya Disease
Source: Transl Stroke Res. 2023 Jun 3;15(4):729–38. doi: 10.1007/s12975-023-01159-z (PMC11226534; doi:10.1007/s12975-023-01159-z)
Supplement: Supplementary file 1 — Online Resource:Supplementary Methods. Supplementary Tables S1–S4. Supplementary Figures S1–S3. Supplementary Table S1 Characteristics of each identified rare variant of RNF213 in this study. Supplementary Table S2 Information on RNF213 p.Ala4399Thr and p.Glu4950Asp. Supplementary Table S3 Clinical manifestations at diagnosis and angiographic features of each hemisphere with p.Ala4399Thr or RV. Supplementary Table S4 Association between angiographic profiles and clinical manifestations at diagnosis. Supplementary Fig. S1 Flowchart of the selection process. Supplementary Fig. S2 Representative angiography of each type of periventricular anastomosis (PA). Supplementary Fig. S3 Kaplan–Meier curves for de novo ischemia and hemorrhage in asymptomatic hemispheres (DOCX 2114 kb) [file 12975_2023_1159_MOESM1_ESM.docx]

**Online Resource**

**Title**: *RNF213* p.Arg4810Lys Wild Type is Associated with De Novo Hemorrhage in Asymptomatic Hemispheres with Moyamoya Disease

Seiei Torazawa^1^, MD; Satoru Miyawaki^1^, MD, PhD; Hideaki Imai^1,2^, MD, PhD; Hiroki Hongo^1^, MD, PhD; Daiichiro Ishigami^1^, MD, PhD; Masahiro Shimizu^3^, MD, PhD; Hideaki Ono^1,4^, MD, PhD; Yuki Shinya^1^, MD, PhD; Daisuke Sato^1^, MD; Yu Sakai^1^, MD; Motoyuki Umekawa^1^, MD; Satoshi Kiyofuji^1^, MD, PhD; Daisuke Shimada^6^, MD, PhD; Satoshi Koizumi^1^, MD; Daisuke Komura^5^, MD, PhD; Hiroto Katoh^5^, MD, PhD; Shumpei Ishikawa^5^, MD, PhD; Hirofumi Nakatomi^6^, MD, PhD; Akira Teraoka^7^, MD, PhD; Nobuhito Saito^1^, MD, PhD

^1^ Department of Neurosurgery, Faculty of Medicine, The University of Tokyo

^2^ Department of Neurosurgery, Tokyo Shinjuku Medical Center

^3^ Department of Neurosurgery, Kanto Neurosurgical Hospital

^4^ Department of Neurosurgery, Fuji Brain Institute and Hospital

^5^ Department of Preventive Medicine, Graduate School of Medicine, The University of Tokyo

^6^ Department of Neurosurgery, Kyorin University Hospital

^7^ Department of Neurosurgery, Teraoka Memorial Hospital

**Corresponding Author:** Satoru Miyawaki, MD, PhD (ORCID: 0000-0001-6369-3391)

Department of Neurosurgery, The University of Tokyo Hospital,

7-3-1 Hongo, Bunkyo-ku, Tokyo 113-8655, Japan

Telephone number: +81-3-5800-8853

Fax number: +81-3-5800-8655

E-mail: [smiya-nsu@m.u-tokyo.ac.jp](mailto:smiya-nsu@m.u-tokyo.ac.jp)

**Supplementary Methods**

**Diagnostic Criteria for Quasi-Moyamoya Disease [1]**

Patients with Moyamoya syndrome associated with the following diseases were diagnosed with the quasi-moyamoya disease and excluded from this study.

1. Autoimmune diseases (systemic lupus erythematosus, antiphospholipid syndrome, polyarteritis nodosa, Sjögren syndrome, etc.); 2. Meningitis, 3. Brain tumors, 4. Down’s syndrome, 5. Neurofibromatosis type 1, and 6. Cerebrovascular lesions after head irradiation.

**Diagnostic Criteria for Each Underlying Disease**

The diagnostic criteria for each underlying disease were as follows: hypertension; systolic blood pressure ≥ 140 mmHg or the use of antihypertensive drugs: diabetes mellitus; fasting blood glucose > 126 mg/dL, occasional blood glucose > 200 mg/dL, or the use of hypoglycemic drugs: dyslipidemia; low-density lipoprotein cholesterol in fasting blood ≥ 140 mg/dL, high-density lipoprotein cholesterol < 40 mg/dL, triglyceride level > 150 mg/dL, or oral treatment with lipid-lowering drugs.

**Reference**

1. Yamamoto S, Hori S, Kashiwazaki D, Akioka N, Kuwayama N, Kuroda S. Longitudinal anterior-to-posterior shift of collateral channels in patients with moyamoya disease: an implication for its hemorrhagic onset. J Neurosurg. 2018;130:884-90. <https://doi.org/10.3171/2017.9.JNS172231>

**Supplementary Tables**

**Supplementary Table S1** Characteristics of each identified rare variant of *RNF213* in this study

| Position (GRCh38) | rsID | Amino Acid Change | AF in gnomAD | AF in ToMMo | SIFT | Polyphen2 | CADD  score |
| --- | --- | --- | --- | --- | --- | --- | --- |
| 17:80287908 | rs775609914 | p.His119Tyr | 2.0*10^-5^ | 8.1*10^-4^ | Tolerated | Benign | 0.07 |
| 17:80288310 | rs140369116 | p.Pro253Ser | 3.0*10^-4^ | 1.8*10^-3^ | Deleterious | Possibly damaging | 8.7 |
| 17:80291683 | rs199729731 | p.His443Asp | 2.6*10^-5^ | 1.5*10^-3^ | Tolerated | Benign | 7.1 |
| 17:80325072 | rs777013389 | p.Arg1023Trp | 8.5*10^-5^ | 3.5*10^-4^ | Tolerated | Benign | 5.1 |
| 17:80343161 | rs369925564 | p.Asp2007Asn | 2.6*10^-5^ | 3.4*10^-3^ | Tolerated | Possibly damaging | 4.0 |
| 17:80345654 | rs761027115 | p.Gly2440Asp | 3.3*10^-5^ | 1.3*10^-3^ | Tolerated | Benign | 22.5 |
| 17:80346446 | rs146486225 | p.Arg2704Gln | 6.6*10^-6^ | 3.5*10^-5^ | Tolerated | Benign | 23.3 |
| 17:80346461 | rs185670413 | p.Arg2709Thr | 8.5*10^-5^ | NA | Deleterious | Benign | 0.18 |
| 17:80347516 | rs1260548587 | p.Glu3061Lys | 6.6*10^-6^ | 1.4*10^-4^ | Tolerated | Benign | 0.17 |
| 17:80358422 | rs375097553 | p.Met3666Thr | 1.6*10^-4^ | NA | Deleterious | Damaging | 25.5 |
| 17:80368031 | rs766766115 | p.Val4015Met | 1.3*10^-5^ | NA | Deleterious | Damaging | 20.4 |
| 17:80372731 | rs138029774 | p.Pro4250Thr | 3.1*10-^3^ | 5.8*10^-3^ | Tolerated | Benign | 1.0 |
| 17:80386819 | rs371441113 | p.Glu4950Asp | 9.9*10^-5^ | 1.4*10^-4^ | Tolerated | Damaging | 14.3 |
| 17:80389879 | NA | p.Ser5083Ala | NA | NA | Tolerated | Possibly damaging | 24.3 |

AF, allele frequency; gnomAD, Genome Aggregation Database (v.3.1.2); ToMMo, Tohoku Medical Megabank Organization (ToMMo 14KJN); SIFT, Sorting Intolerant from Tolerant; CADD, combined annotation-dependent depletion (GRCh38-v1.6); NA, not applicable

**Supplementary Table S2** Information on *RNF213* p.Ala4399Thr and p.Glu4950Asp

| Variant | AF in gnomAD | AF in ToMMo | SIFT | Polyphen2 | CADD | Associated phenotype in previous reports | Number of cases in this cohort  n (%) | MAF  in this cohort | Phenotype  in this cohort (ischemia/hemorrhage)  n (%) |
| --- | --- | --- | --- | --- | --- | --- | --- | --- | --- |
| p.Ala4399Thr | 8.7*10^-3^ | 5.9*10^-2^ | Tolerated | Possibly damaging | 13.6 | Hemorrhage (OR=2.8) | 17 (12.2) | 4.3*10^-2^ | 9 (52.9) / 7 (41.2) |
| p.Glu4950Asp | 9.9*10^-5^ | 1.4*10^-4^ | Tolerated | Damaging | 14.3 | Ischemia (OR=2.2) | 1 (0.7) | 3.6*10^-3^ | 1 (100) / 0 (0) |

AF, allele frequency; gnomAD, Genome Aggregation Database (v.3.1.2); ToMMo, the database of Tohoku Medical Megabank Organization (ToMMo 14KJN); SIFT, Sorting Intolerant from Tolerant; CADD, combined annotation-dependent depletion (GRCh38-v1.6); n, number.

**Supplementary Table S3** Clinical manifestations at diagnosis and angiographic features of each hemisphere with p.Ala4399Thr or RV

|  | All hemispheres  (n = 253) | | p.Ala4399Thr  (n = 31) | | | RV  (n = 28) | | |
| --- | --- | --- | --- | --- | --- | --- | --- | --- |
|  | n | % | n | % | *P* value | n | % | *P* value |
| **Symptoms at diagnosis** |  |  |  |  |  |  |  |  |
| Asymptomatic | 124 | 49.0 | 17 | 54.8 | 0.488 | 12 | 42.9 | 0.490 |
| Ischemia | 103 | 40.7 | 9 | 29.0 | 0.158 | 13 | 46.4 | 0.514 |
| Hemorrhage | 26 | 10.3 | 5 | 16.1 | 0.197 | 3 | 10.7 | 0.571 |
| **Angiographical features** |  |  |  |  |  |  |  |  |
| Lenticulostriate PA | 58 | 22.9 | 6 | 19.4 | 0.614 | 4 | 14.3 | 0.249 |
| Choroidal PA | 88 | 34.8 | 11 | 35.5 | 0.930 | 11 | 39.3 | 0.596 |
| Thalamic PA | 24 | 9.5 | 4 | 12.9 | 0.336 | 1 | 3.6 | 0.224 |
| PCA involvement | 34 | 13.4 | 2 | 6.5 | 0.176 | 4 | 14.3 | 0.539 |
| Suzuki grade, median (IQR) | 3 (3-3) | | 3 (3-3) | | 0.440 | 3 (3-3) | | 0.848 |

RV, rare variant; IQR, interquartile range; PA, periventricular anastomosis, PCA; posterior cerebral artery

**Supplementary Table S4** Association between angiographic profiles and clinical manifestations at diagnosis

|  | Ischemia (Yes, n = 103) | | | Hemorrhage (Yes, n = 26) | | |
| --- | --- | --- | --- | --- | --- | --- |
|  | Yes, n (%) | No, n (%) | *P* value, OR (95% CI) | Yes, n (%) | No, n (%) | *P* value, OR (95% CI) |
| **Angiographical features** |  |  |  |  |  |  |
| Lenticulostriate PA | 23 (22.3) | 35 (23.3) | *p* = 0.852, 0.95 (0.52-1.72) | 7 (26.9) | 51 (22.5) | *p* = 0.609, 1.27 (0.51-3.19) |
| Choroidal PA | 34 (33.0) | 54 (36.0) | *p* = 0.624, 0.88 (0.52-1.49) | 15 (57.7) | 73 (32.2) | ***p* = 0.010, 2.88 (1.26-6.57)** |
| Thalamic PA | 8 (7.8) | 16 (10.7) | *p* = 0.439, 0.71 (0.29-1.72) | 5 (19.2) | 19 (8.4) | *p* = 0.083, 2.61 (0.88-7.70) |
| PCA involvement | 16 (15.5) | 18 (12.0) | *p* = 0.418, 1.35 (0.65-2.79) | 4 (15.4) | 30 (13.2) | *p* = 0.474, 1.19 (0.39-3.71) |
| Suzuki grade |  |  | ***p* = 0.002, 1.87 (1.27-2.76)** |  |  | ***p* = 0.042, 1.89 (1.03-3.49)** |
| 1 | 1 (1.0) | 18 (12.0) |  | 0 (0.0) | 19 (8.4) |  |
| 2 | 7 (6.8) | 17 (11.3) |  | 0 (0.0) | 24 (10.6) |  |
| 3 | 83 (80.6) | 98 (65.3) |  | 22 (84.6) | 159 (70.0) |  |
| 4 | 9 (8.7) | 15 (10.0) |  | 4 (15.4) | 20 (8.8) |  |
| 5 | 3 (2.9) | 1 (0.7) |  | 0 (0.0) | 4 (1.8) |  |

CI, Confidence interval; PA, periventricular anastomosis, PCA; posterior cerebral artery

**Supplementary Figures and Figure Legends**


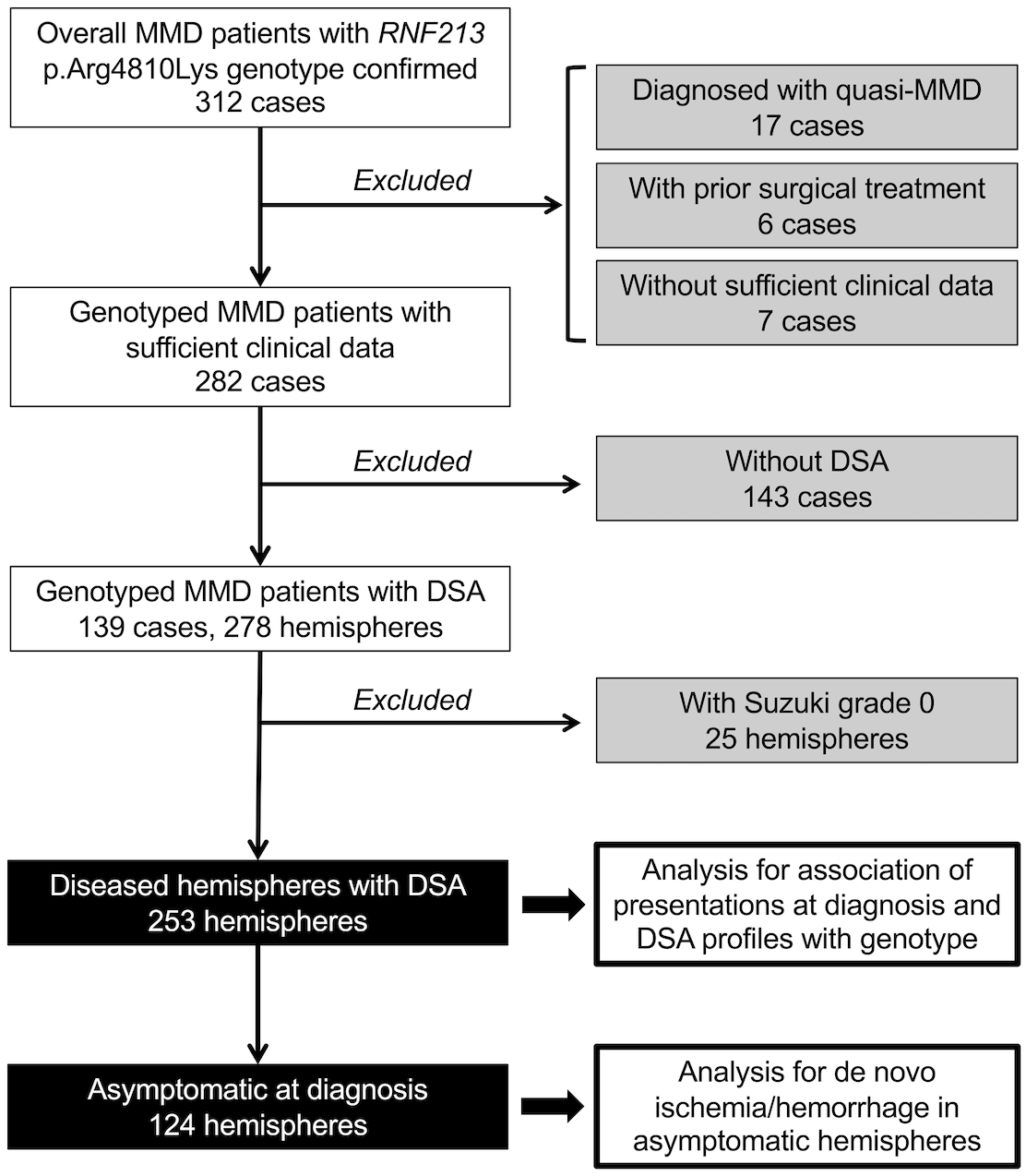


**Supplementary Fig. S1** Flowchart of the selection process

The process for selecting cases with Moyamoya disease (MMD) and hemispheres for analysis. Among the 312 patients with a verified *RNF213* p.R4810K genotype diagnosed with MMD, 17 were diagnosed with quasi-MMD, six had prior surgical treatment, and seven had insufficient clinical data. After excluding these patients and those without digital subtraction angiography (DSA), 139 patients who underwent DSA at diagnosis were enrolled. We excluded hemispheres with Suzuki grade 0 and analyzed the remaining 253 hemispheres. Among these, 124 asymptomatic hemispheres were analyzed for de novo stroke events at diagnosis.


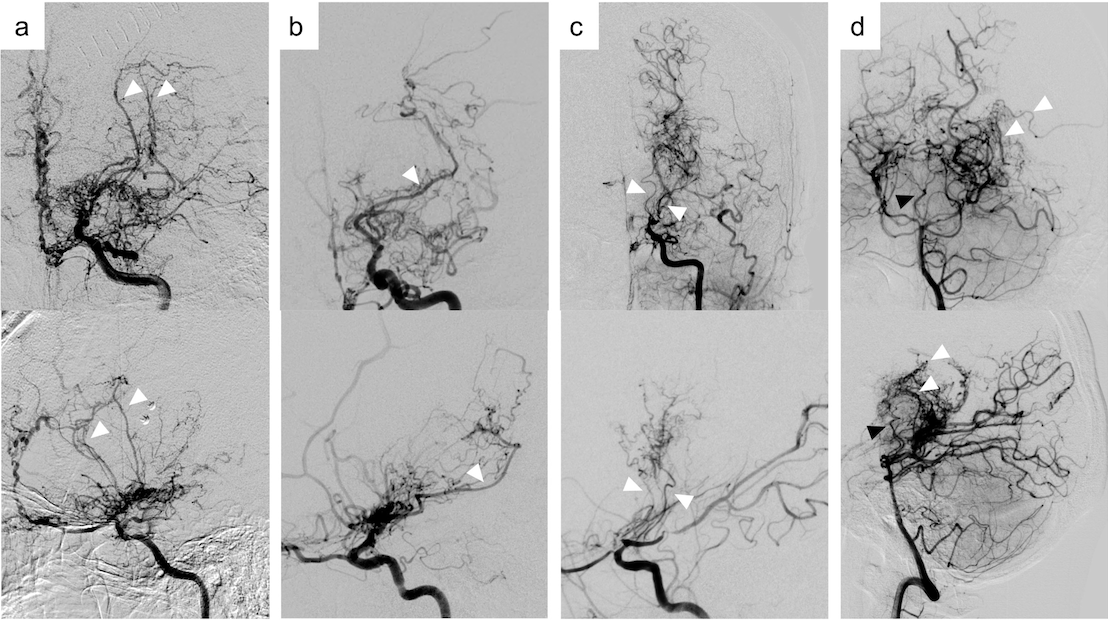


**Supplementary Fig. S2** Representative angiography of each type of periventricular anastomosis (PA)

Representative images of cerebral digital subtraction angiography at diagnosis. The upper row shows the anterior-posterior view, and the lower row shows the lateral view of the same patient.

a: Lenticulostriate PA originating from the lenticulostriate arteries connecting the medullary artery to the anterior periventricular area.

b: Choroidal PA arising from the anterior choroidal artery connecting the medullary artery to the posterior periventricular area.

c: Thalamic PA of the thalamotuberal arteries from the posterior communicating artery extending beyond the thalamus and connecting to the medullary artery.

d: Thalamic PA of the thalamogeniculate artery (white arrowhead), extending laterally beyond the thalamus and connecting to the insular artery. The thalamoperforating artery (black arrowhead) was also well developed.


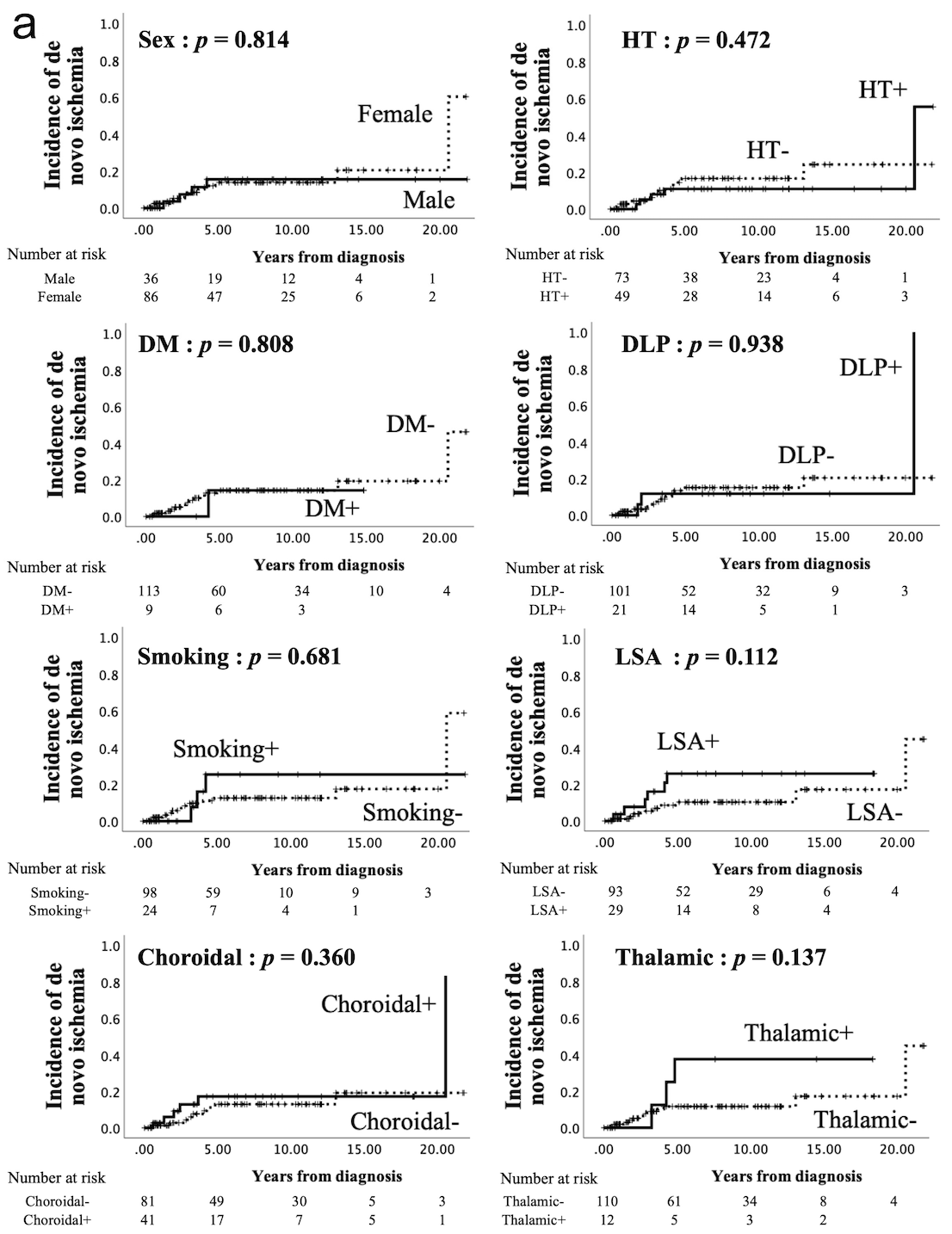


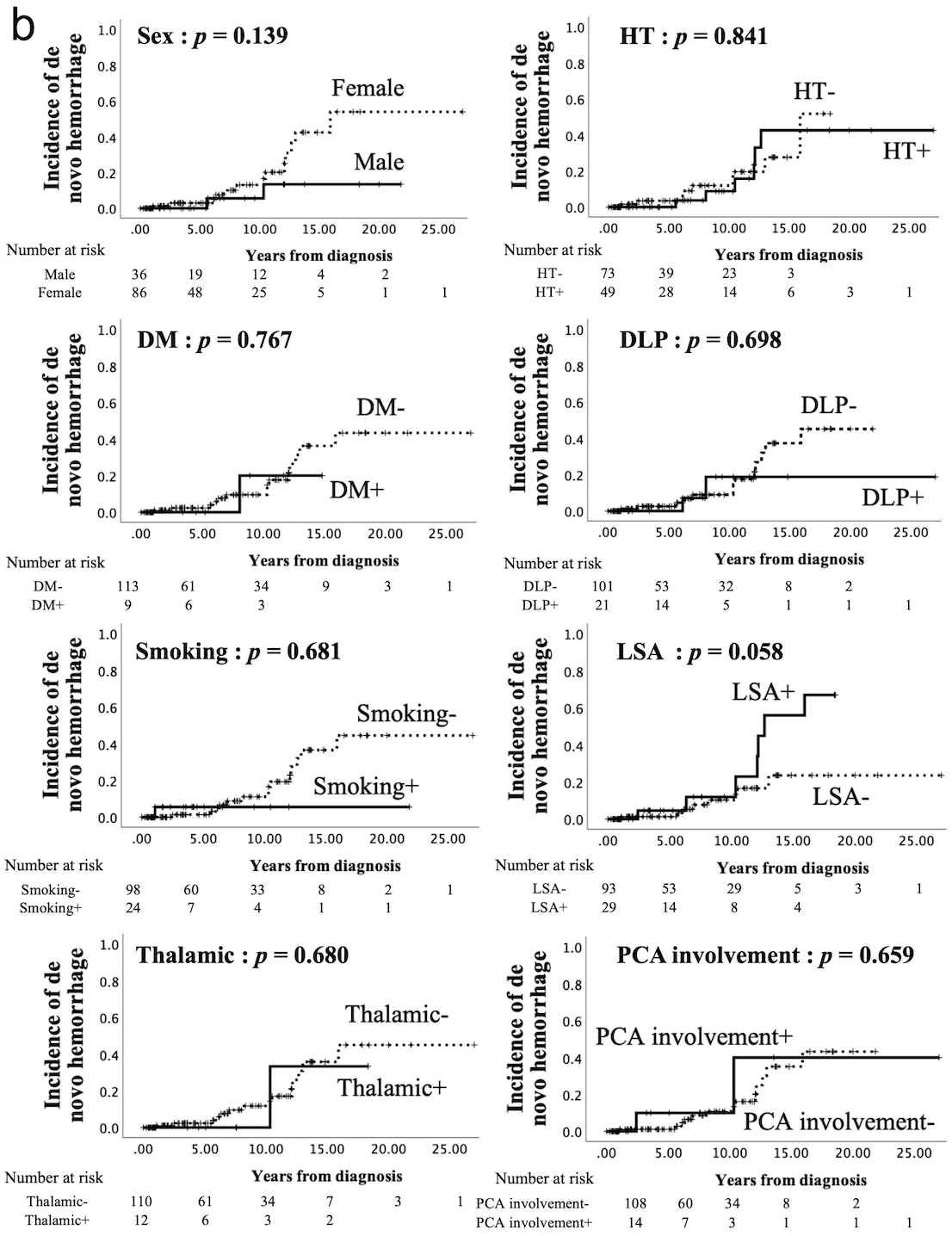


**Supplementary Fig. S3** Kaplan–Meier curves for de novo ischemia and hemorrhage in asymptomatic hemispheres

Kaplan–Meier curves for de novo ischemia (a) and de novo hemorrhage (b) in asymptomatic hemispheres. *P*-values were calculated using the log-rank tests.

DLP, dyslipidemia; DM, diabetes mellitus; HT, hypertension; LSA, lenticulostriate artery; PCA; posterior cerebral artery
